# Brown and Red Seaweeds Serve as Potential Efflux Pump Inhibitors for Drug-Resistant* Escherichia coli*

**DOI:** 10.1155/2019/1836982

**Published:** 2019-01-01

**Authors:** Wen-Jung Lu, Hsuan-Ju Lin, Pang-Hung Hsu, Margaret Lai, Jen-Yu Chiu, Hong-Ting Victor Lin

**Affiliations:** ^1^Department of Food Science, National Taiwan Ocean University, No. 2, Pei-Ning Road, Keelung 202, Taiwan; ^2^Department of Bioscience and Biotechnology, National Taiwan Ocean University, No. 2, Pei-Ning Road, Keelung 202, Taiwan; ^3^Center of Excellence for the Oceans, National Taiwan Ocean University, No. 2, Pei-Ning Road, Keelung 202, Taiwan

## Abstract

Multidrug-resistant pathogens are a significant clinical problem. Efflux pump inhibitors (EPIs) can restore the activities of existing antibiotics by interfering with drug efflux pumps located in bacterial cell membranes. Seaweeds are important sources of biologically active metabolites of natural origin; however, their potential as EPIs remains uninvestigated. Here, functional extracts from the brown seaweeds* Laminaria japonica* and* Sargassum horneri* and the red seaweeds* Gracilaria *sp. and* Porphyra dentata* were evaluated as potential EPIs against drug-resistant* Escherichia coli*. All these extracts were found to potentiate the activities of drugs in modulation tests, although not to the same extent. Synergistic effects of the extracts and the drug clarithromycin were observed from the onset of Time-kill assays, with no evidence of bacterial regrowth. Ethidium bromide accumulation studies revealed that the efflux decreased in the presence of each extract, as indicated by the presence of EPIs. Most identified EPIs that have been discovered to date have aromatic structures, and the seaweed extracts were found to contain various terpenes, terpenoids, phenolic compounds, indoles, pyrrole derivatives, alkaloids, and halogenated aromatic compounds. Our study highlights the potential of these compounds of the seaweeds as drug EPIs.

## 1. Introduction

Multidrug-resistant (MDR) pathogens are a significant clinical problem. O'Neill [1], commissioned by the British government, estimated that, by 2050, global deaths due to drug-resistant infections will increase from 700,000 to 10 million annually and that the global economic loss may reach US$100 trillion. Attempts by the pharmaceutical industry to reverse this trend have achieved limited success, and the development and release of novel antimicrobial agents have faltered [2, 3]. Efflux pumps in bacteria are major contributors to drug resistance; they extrude a broad spectrum of antibiotics to the exterior of the organism. Hence, infections caused by these pathogens can be difficult to treat [4]. For example, the inner membrane transporter AcrB (resistance-nodulation-division family) is the major MDR efflux pump in* Escherichia coli* and can assemble with a periplasmic adaptor protein AcrA and an outer membrane factor TolC. This assembly confers drug resistance by translocating various types of antibiotics, such as macrolides [5], tetracyclines [6, 7], fluoroquinolones [8], and *β*-lactams [9], across the inner and outer membranes [10–12]. The use of EPIs has been rapidly gaining attention as a novel approach to treat infections caused by pathogens expressing MDR efflux pumps [13, 14]. Their use in adjunctive therapy may restore the activities of existing antibiotics by interfering with efflux pumps, thereby allowing therapeutically ineffective antibiotics to be reintroduced into clinical practice. EPIs may affect the function of efflux pumps by (i) regulating the expression of the pump, (ii) inhibiting the functional assembly of the membrane transporter complex involved in drug efflux, (iii) interfering with the energy required for active drug transport, and (iv) inhibiting drug transport via competitive/noncompetitive binding or by physically blocking the efflux channel [15]. Several classes of EPIs, including antibiotic (tetracyclines, aminoglycosides, and fluoroquinolones) analogs [16–18], amide derivatives (aromatic nitrogen-containing compounds) [19, 20], indoles [21], alkaloids [22–24]), flavonoids [25, 26], aromatic ketones [27], terpenes [28], and oligosaccharides [29], have been reported to date. Several well-known EPIs, such as phenylalanine-arginine *β*-naphthylamide (PA*β*N) [30], carbonyl cyanide* m*-chlorophenylhydrazone (CCCP) [31], verapamil [32], and reserpine [33], have seen limited clinical use and development owing to their toxicity [14]. Plants are a promising source of novel EPIs owing to their low toxicity and compound diversity. In addition, plants are natural, sustainable, and largely unexplored [34]. Capsaicin (8-methyl-N-vanillyl-6-nonenamide), extracted from hot chilies (genus* Capsicum*), has been shown to significantly reduce the minimum inhibitory concentration of ciprofloxacin by targeting the transporter NorA of* Staphylococcus aureus* strains [35]. Piperine, a plant alkaloid found in black pepper (*Piper nigrum*) and long pepper (*Piper longum*) and belonging to the family* Piperaceae*, potentiates the activity of ciprofloxacin against* S. aureus* strains expressing drug efflux pumps [22, 36]. Lanatoside C and daidzein were chosen from a phytochemical database via* in silico* screening. Both compounds were shown to potentiate the activities of levofloxacin and carbenicillin and increase the accumulation of ethidium bromide (EB) in* E. coli *[37]. Seaweeds have been used as food and medicine since centuries. They have a short generation cycle and can be easily cultivated in various aquatic environments [38, 39]. Seaweeds are an important source of biologically active metabolites owing to their availability, diversity, and productivity [40]. They are rich in phenolics [41], terpenoids [42], alkaloids [43], flavonoids [44], and polysaccharides [45], some of which possess specific characteristics that are rare or absent in terrestrial plants. Seaweeds are thought to possess antioxidant [46], antimicrobial [47], anti-inflammation [48, 49], antidiabetic [50, 51], and anticancer activities [52]. Despite these factors, their potential as EPIs has not yet been evaluated. Gram-negative pathogen* E. coli* is one of the most troublesome clinical bacterial species. Moreover, to date, very few EPIs for Gram-negative bacteria have been reported [53]. To our knowledge, this study was the first to evaluate the potential of ethanolic extracts of the brown seaweeds* Laminaria japonica* and* Sargassum horneri* and red seaweeds* Gracilaria *sp. and* Porphyra dentata* as EPIs against drug-resistant* E. coli*.

## 2. Materials and Methods

### 2.1. Preparation of Ethanolic Extracts from Seaweeds

*L. japonica*,* Gracilaria *sp., and* P. dentata *were purchased from a local market in Chao-Ching Park.* S. horneri *was harvested in May 2017 along the north east coast of Taiwan. The seaweeds were washed, air-dried (50°C), ground, and sieved through 0.25 mm pores and stored in a freezer until use. The ethanolic seaweed extracts were obtained by maceration. In brief, the seaweed powders were soaked in 95% ethanol (solid/solvent ratio = 1/10) with slow stirring at room temperature for 24 h for extraction. The mixture was filtered through, and the filtered algal residue was extracted once again using the previous procedure. The ethanolic extracts were obtained by rotary evaporation and lyophilization and stored in dark at -20°C until use.

### 2.2. Bacterial Strains, Media, and Chemicals

*E*.* coli *Kam3 (DE3) which is the* acrB* deletion strain was used in drug susceptibility, modulation and drug accumulation assays [54]. The bacteria were grown in Luria-Bertani broth (LB) and Mueller-Hinton broth (MH broth) for cultivation and broth microdilution experiments.

### 2.3. Cloning of E. coli Efflux Pump AcrB

*acrB* gene was cloned from the* E. coli *K12 chromosome by using PCR method. The* acrB* gene amplified by using primers 5′-AAAACCCATATG1CCTAATTTCTTTATCGATCGCC-3′ and 5′-AAAACCGTCGAC2TCAATGATGATCGACAGTAT-3′ which was digested with NdeI and XhoI restriction enzymes and inserted onto pSYC vector (pQE100 derivative, T5 promoter) at the NdeI-XhoI site. The pSYC plasmid encoding* acrB* was transform into* E. coli* Kam3 (DE3) in drug susceptibility, modulation, and drug accumulation assays. The ampicillin (100 *μ*g/mL) was used in the experiments.

### 2.4. IC_*50*_ and Modulation Tests

The IC_50_ experiments and modulation tests were carried out as previously described with some modifications [55]. The IC_50_ of the antibiotic erythromycin, clarithromycin, and tetracycline, and the ethanolic seaweed extracts against drug-sensitive and drug-resistant* E. coli *strains were determined by using microdilution methods (MH broth), individually, with an inoculum of logarithmic-phase cells (cell density of an OD600 of 0.05 to 0.1). For the modulation tests, the IC_50_ of the antibiotic erythromycin, clarithromycin, and tetracycline were determined in the presence of 1/2 or 1/4 IC_50_ of the seaweed extracts, individually. 500 *μ*g/mL was chosen as the IC_50_ concentration of the seaweed extracts in the modulation assays when the IC_50_ > 500 *μ*g/mL.

### 2.5. Time-Kill Assays

The Time-kill experiments were carried out according to a previous study [27], with some modifications. Time-kill study of clarithromycin (1/2 IC_50_) alone or in the presence of seaweed extract (IC_50_, 1/2 IC_50_ or 1/4 IC_50_) was performed in 50 ml volume conical flasks containing 20 ml* E. coli cells* (7 Log CFU/mL). 500 *μ*g/mL were chosen as the IC_50_ concentration for the seaweed extracts in the Time-kill assays when the IC_50_ > 500 *μ*g/mL. Each analysis was done in triplicate with a control without seaweed extract.

### 2.6. EB Accumulation Assay

The EB accumulation assay was performed according to previous studies [56, 57], with the following modifications. The* E. coli* cells were grown to mid-log phase in MH broth and collected by centrifugation (5000 ×* g*, 5 min and 4°C). The cells were resuspended twice in phosphate buffered saline (PBS) (10 mM Na_2_HPO_4_, 1.8 mM KH_2_PO_4_, 137 mM NaCl, 2.7 mM KCl, 1 mM CaCl_2_, and 0.5 mM MgCl_2_ at pH 7.4) and diluted in PBS in a final OD600 of 0.5. The cell suspension was incubated in 96 well plate with the filter-sterilized glucose to a final concentration of 25 mM at room temperature for 3 min. The EB was added to a final concentration of 25 *μ*M and the fluorescence was measured over 38 min using at excitation and emission wavelengths of 520 nm and 600 nm. The effects of CCCP (final concentration of 20 *μ*g/mL) and algal ethanolic extracts (1/2 IC_50_), individually, were added to bacterial suspension before the fluorescence was measured.

### 2.7. Gas-Chromatography-Mass Spectrometry

Gas Chromatography tandem Mass Spectrometry (GC-MS/MS) was performed by a PolarisQ Ion Trap GC-MS/MS system with a split/splitless injector and Equity®-5 capillary GC column (L × I.D. 30 m × 0.25 mm, d_f_ 0.25 *μ*m). The carrier gas used was Helium (He) at a flow rate of 1 ml/min, and the injection port was maintained at 280°C. The temperature programming at 50°C for 2 min and increased at a rate of 15°C/min until 250°C and held at 250°C for 2 min. Electron impact ionization (EI) of the GC column eluents screening ranges from 100 to 1000 m/z, and the mass spectrometry data was analyzed by database provided by National Institute of Standards and Technology.

### 2.8. Statistical Analysis

Data are analyzed statistically by using SPSS version 12 (Chicago, IL, USA) and presented as means ± standard deviation. One-way analysis of variance (ANOVA) was used to determine statistical differences between samples means, with the level of significance set at* p* < 0.05, and multiple comparisons of means were accomplished by Tukey test.

## 3. Results and Discussion

### 3.1. Ethanol Extraction of the Four Seaweeds and Their IC_*50*_ against Drug-Susceptible and Drug-Resistant E. coli

The chemical structures of several EPIs derived from natural sources have been identified. These include the alkaloids reserpine [58] and piperine [22], the flavonolignans 5′-methoxyhydnocarpin [59] and silybin [60], the flavones baicalein [61] and chrysosplenol-D [62], and the diterpene carnosol [63]. These compounds were initially extracted using organic solvents owing to their lipophilic nature. We used ethanol to extract potential EPIs from* L*.* japonica*,* S. horneri, Gracilaria *sp., and* P. dentata* and obtained yields of 5.2%, 4.3%, 6.3%, and 3.9%, respectively (Table 1). This is in agreement with the findings of Dickson et al. [64], who used ethanol to extract drug-potentiating substances from the terrestrial plants* Microglossa pyrifolia*,* Mezoneuron benthamianum*, and* Securinega virosa*; they obtained yields of 5.9%, 4.3%, and 3.6%, respectively.

The ethanolic extracts were tested using the microdilution method against drug-susceptible (Kam3) and drug-resistant (Kam-AcrB)* E. coli *strains based on their IC_50_ measurements. As shown in Table 1, the IC_50_ values of the* L*.* japonica*,* S. horneri*,* Gracilaria *sp., and* P. dentata *extracts against Kam3 were 125, 250, 62.5, and 250 *μ*g/mL, respectively, thereby indicating that the extracts might contain some antibacterial compounds. The naturally occurring aromatic organic compound p-cymene found in some algal extracts [65] has been shown to exhibit antibacterial activity against* E. coli *O157: H7 [66].

Interestingly, the ethanolic extracts at the tested concentrations showed no inhibitory effects against the drug-resistant* E. coli *strain. The IC_50_ values of all the four extracts against Kam3-AcrB were >500 *μ*g/mL, indicating that the antibacterial substances in the extracts are expelled by the multidrug transporter AcrB. Lomovskaya et al. [30] reported that Mex pumps in* Pseudomonas aeruginosa* confer resistance to EPI PA*β*N, thereby indicating that PA*β*N is efficiently extruded.

### 3.2. Seaweed Extracts Potentiate the Activities of Macrolides against Drug-Susceptible and Drug-Resistant E. coli

AcrAB-TolC in* E. coli *is the most studied tripartite pump to date; it is linked to a wide range of drugs, including macrolides [5], tetracyclines [6, 7], fluoroquinolones [8], and *β*-lactams [9], and dyes, such as EB [67]. The four ethanolic extracts were added at subinhibitory concentrations of 1/2 and 1/4 IC_50_ in the modulation assays with the macrolides erythromycin and tetracycline, both of which are known to be substrates of the RND drug transporter AcrB.

As shown in Table 2, the extracts from brown seaweeds* L. japonica* and* S. horneri* and red seaweeds* Gracilaria *sp. at a concentration of 1/2 IC_50_ were able to potentiate the activity of erythromycin against the Kam3 strain, with a modulation factor of 4, and the* S. horneri *and* Gracilaria* sp. extracts were even found to have potentiating activities at 1/4 IC_50_. In addition, the* S. horneri *and* Gracilaria* sp. extracts were able to potentiate the activity of erythromycin against the Kam3-AcrB strain, with a modulation factor of 8 and 2 at 1/2 IC_50_, respectively, and 2 at 1/4 IC_50_. Intriguingly, the potentiating activities of the extracts were not observed in the modulation assays using tetracycline (data not shown); this antibiotic shares its mechanism of action (i.e., protein synthesis inhibition) with erythromycin. Lomovskaya et al. [30] demonstrated that PA*β*N did not potentiate the activities of all antibiotic substrates of the MexAB-OprM efflux pump to the same extent. They speculated that different antibiotics may have different binding sites on the pump and that PA*β*N-induced inhibition is binding site specific.

We further investigated the potentiating activities of the extracts with another macrolide clarithromycin in the modulation assay (Table 3). The extracts from* L. japonica*,* S. horneri*, and* Gracilaria *sp. at 1/2 IC_50_ exhibited potentiating activities with clarithromycin against Kam3 (modulation factor, 8). In addition, all the extracts were found to potentiate the activity of clarithromycin against Kam3-AcrB at 1/2 and 1/4 IC_50_. The extract from* S. horneri* appeared to possess the greatest potentiating activity (modulation factor, 16 at 1/2 IC_50_).

### 3.3. Effect of Seaweed Extracts on Time-Kill Curves

**Figure 1:**
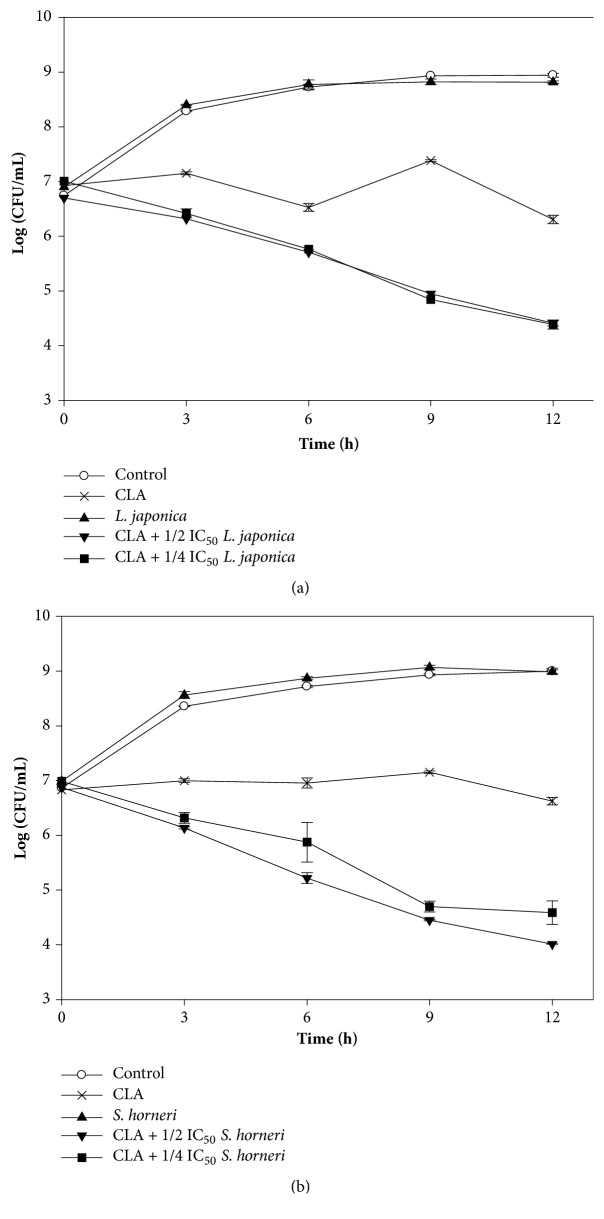
Time-kill curves of clarithromycin alone and combined with brown seaweed (a)* L. japonica* extract and (b)* S. horneri *extract against drug-resistant* E. coli*. The Kam3-AcrB* E. coli* cells at a cell density of 7 Log CFU/mL were added with clarithromycin alone or combined with seaweed extracts, and the cell numbers were monitored every 3 h for 12 h.

To determine the synergistic effect of the seaweed extracts with macrolide over time, the growth of Kam3-AcrB was monitored in the presence of clarithromycin and clarithromycin + a seaweed extract.  Figure 1(a) indicates that Kam-AcrB was allowed to grow to log phase and then exposed to clarithromycin, the* L. japonica* extract, or the clarithromycin +* L*.* japonica *extract. The bacteria exposed to the extract alone showed a growth pattern similar to the control group (no addition), whereas the cells exposed to clarithromycin gradually decreased in number.

Furthermore, the number of viable cells exposed to clarithromycin +* L. japonica* extract (1/2 and 1/4 IC_50_) sharply decreased from 7 to 4.4 log CFU/mL after 12 h of incubation. Similar results were observed in the assays with* S. horneri* (Figure 1(b)). Inhibitory effects were not observed when testing the bacteria with* S. horneri* alone. Moreover, the addition of the clarithromycin +* S. horneri* extract (1/2 and 1/4 IC_50_) resulted in a greater inhibitory effect than that of clarithromycin alone. The approximate reduction in cell number was 7 to 4 log CFU/mL after 12 h (1/2 IC_50_).

**Figure 2:**
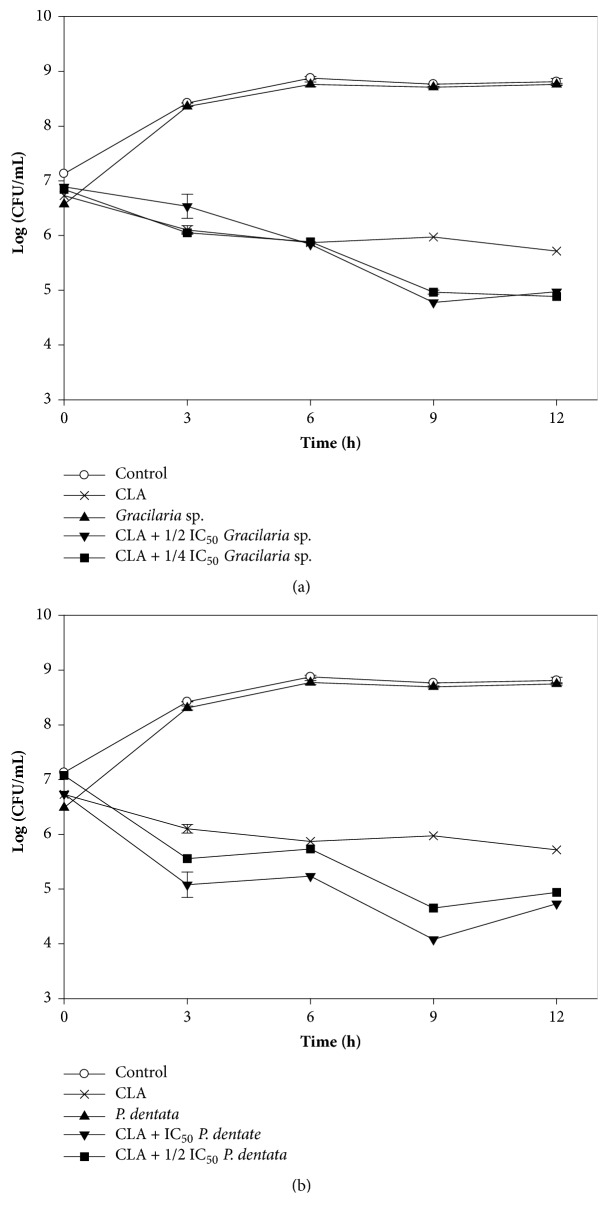
Time-kill curves of clarithromycin alone and combined with red seaweed (a)* Gracilaria *sp. extract and (b)* P. dentata *extract against drug-resistant* E. coli*. The Kam3-AcrB* E. coli* cells at a cell density of 7 Log CFU/mL were added with clarithromycin alone or combined with seaweed extracts, and the cell numbers were monitored every 3 h for 12 h.

We also investigated the synergistic inhibitory effects of clarithromycin and red seaweed extracts against Kam3-AcrB over time.  Figure 2 shows that the addition of* Gracilaria* sp. or* P. dentata* extracts to the cells in the presence of clarithromycin resulted in greater inhibition than when clarithromycin was used alone. Our data suggest that the extracts had synergistic effects on clarithromycin from the onset of incubation, with no bacterial regrowth occurring. Zhou et al. [68] reported similar results of Time-kill assays, thus demonstrating the synergistic antibacterial effects of the alkaloid EPI berberine and the antibiotic ciprofloxacin in a MDR clinical isolate of* Klebsiella pneumoniae*.

### 3.4. Efflux-Mediated Properties of the Seaweed Extracts

**Figure 3:**
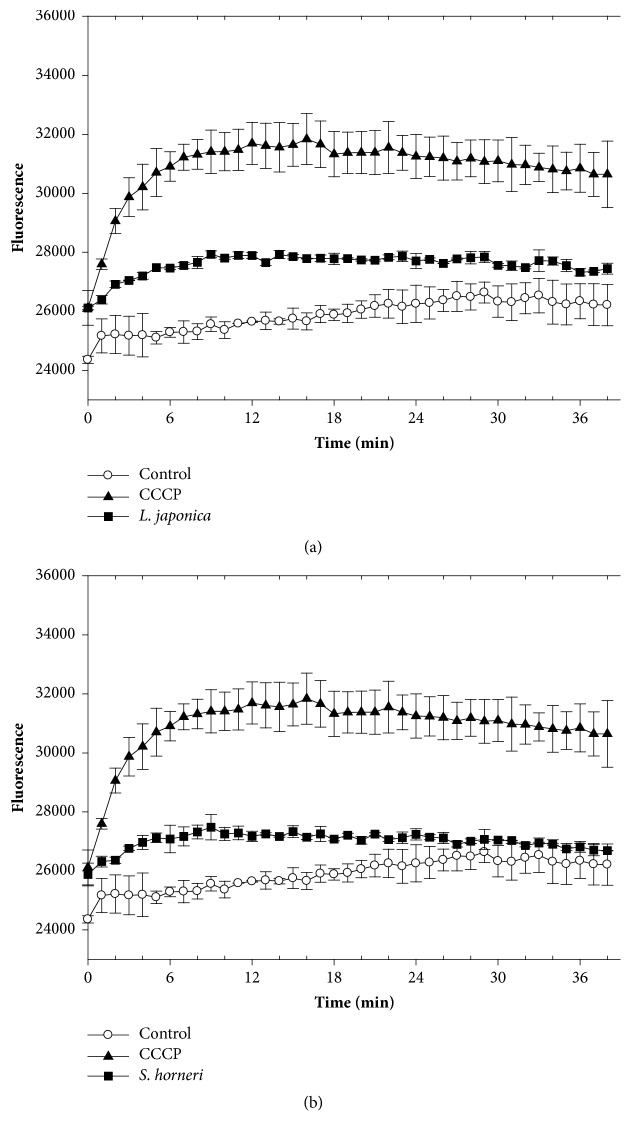
Effects of brown seaweeds (a)* L. japonica* extract and (b)* S. horneri *extract on EB accumulation in drug-resistant* E. coli*. The* E. coli* Kam3-AcrB cells were added with glucose (25 mM) and EB (25 *μ*M) in presence or absence of CCCP (20 *μ*g/mL) or seaweed extracts (1/2 IC_50_). The fluorescence was monitored at Ex 520 nm and Em 600 nm.

**Figure 4:**
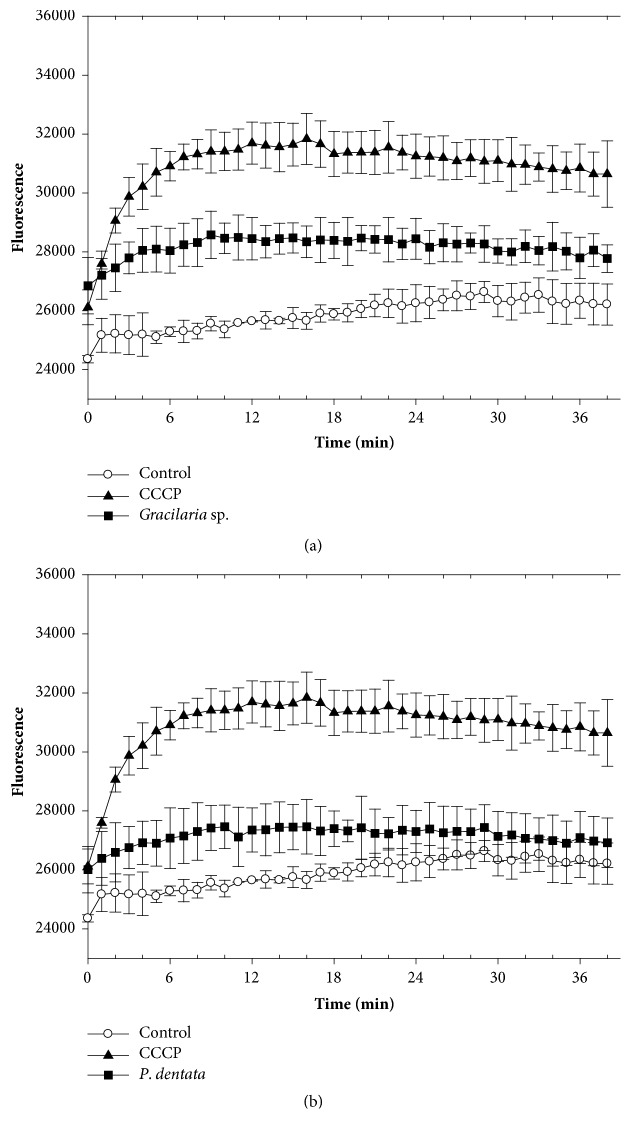
Effects of red seaweeds (a)* Gracilaria* sp. extract and (b)* P. dentata* extract on EB accumulation in drug-resistant* E. coli*. The* E. coli* Kam3-AcrB cells were added with glucose (25 mM) and EB (25 *μ*M) in presence or absence of CCCP (20 *μ*g/mL) or seaweed extracts (1/2 IC_50_). The fluorescence was monitored at Ex 520 nm and Em 600 nm.

To analyze the potential efflux-mediated properties of the seaweed extracts, we monitored the accumulation of EB into Kam3-AcrB in their presence. EB is a substrate of the multidrug transporter AcrB in* E. coli* [67] and intensely fluoresces when bound to DNA.  Figure 3(a) shows that EB accumulation was greater in cells exposed to the extract and positive control CCCP (20 *μ*g/mL; a proton uncoupler) rather than the control (no addition). This indicates that the* L. japonica *extract possesses an efflux pump inhibitor that interferes with EB efflux from Kam3-AcrB. Similar results were observed in cells exposed to* S. horneri *(Figure 3(b)),* Gracilaria* sp. (Figure 4(a)), and* P. dentata *extracts (Figure 4(b)). The EB accumulation assays were also performed in Kam3 in the presences of the seaweed extracts and observed no obvious increase of EB accumulation (data not shown). This might suggest that the seaweed extracts increase the EB accumulation possibly by mediating efflux pump rather than by increasing membrane permeability. Our data indicate that all the extracts contain functional compounds that increase EB accumulation in Kam-AcrB. This highlights their potential as EPIs of AcrB, which is known to be overexpressed with a strong promoter in Kam3-AcrB strains. However, we were unable to rule out the possibility that the extracts interfere with other efflux pumps that translocate EB.

### 3.5. Chemical Compositions of the Seaweed Extracts

Table 4 shows the chemical composition of the seaweed extracts as determined using gas chromatography-mass spectrometry (GC-MS). The classes with the symbol ⌜++⌟ have more chemicals than the classes with the symbol ⌜+⌟ in each seaweed extract. All the extracts contained terpenes, terpenoids, and phenolic compounds. The* S. horneri *and* P. dentata* extracts contained indoles, whereas the* Gracilaria *sp. extract contained pyrrole derivatives. In addition, halogenated aromatic compounds and alkaloids were identified in the red seaweeds.

The chemical structure of several plant-derived and synthetic EPIs has been characterized. The diterpenes carnosol and carnosic acid obtained from the herb* Rosmarinus officinalis* are known to potentiate erythromycin and tetracycline against* S. aureus* strains expressing Tet(K) and Msr (A) pumps [63]. Lorenzi et al. [69] indicated that the monoterpene geraniol obtained from* Helichrysum italicum* increases the efficacy of quinolones and chloramphenicol in* E. coli*. Synthetic indole derivatives, such as [4-benzyloxy-2-(5-nitro-1H-2-yl)-phenyl]-methanol, have been reported as EPIs for the efflux pump NorA in* S. aureus* [70].

Furthermore, synthetic halogenated phenothiazine derivatives, such as chlorpromazine, are thought to inhibit drug efflux pumps via various mechanisms [71]. Dwivedi et al. [23] indicated that alkaloid chanoclavine isolated from* Ipomoea muricata* potentiates the activity of tetracycline against MDR clinical* E. coli* isolate, possibly by inhibiting drug efflux and downregulating the expression of drug transporters. In addition, catharanthine isolated from the leaves of flowering plant* Catharanthus roseus* has been shown to potentiate the activity of tetracycline, possibly due to the inhibition of the efflux pumps in* P. aeruginosa* [24]. 3,4-Dibromopyrrole-2,5-dione, a bacterial halogenated metabolite, is an effective EPI against bacterial strains that overexpress AcrB-TolC, MexAB-OprM, and MexXY-OprM [72]. The plant-derived alkaloids reserpine and piperine are also effective EPIs for fluoroquinolones in* S. aureus* [22, 33]. EPIs hinder the functions of efflux pumps via several mechanisms.

Seaweeds are rich in bioactive terpenes and aromatic compounds, some of which possess specific characteristics that are absent or rarely found in terrestrial plants. For instance, approximately 25% of all halogenated natural products are alkaloids, and most of them are found in marine organisms [73]. Halogenated compounds obtained from seaweeds are varied and range from indoles, terpenes, and phenols to halogenated hydrocarbons; investigations into their potential use as EPIs would be beneficial [74]. Our IC_50_ data in Table 1 indicated that some antibacterial substances in the extracts are expelled by the multidrug transporter AcrB, which might suggest that the inhibition of drug transport was a result of competitive binding to AcrB. Further chromatographic purification of the ethanolic extracts obtained from seaweeds is required to identify and produce pure, active compounds. The findings of this study should encourage further research into seaweeds and their extracts with a view to identifying new EPIs with potential clinical applications.

## 4. Conclusions

The ethanolic seaweed extracts were able to potentiate the activities of macrolides against* E. coli* and inhibit the action of efflux pumps in clinically important pathogens, thereby highlighting their potential as effective EPIs. We believe these extracts should be further investigated to exploit their ability to block drug efflux pumps, thereby facilitating the development of novel antimicrobial agents effective against clinically important MDR pathogens.

## Figures and Tables

**Table 1:** Ethanol extraction yield of the seaweed extracts and their IC_50_ against drug-susceptible and drug-resistant *E. coli*.

Macroalgae	Extraction yield (%)	IC_50_ (*μ*g/mL)	
		Kam3	Kam3-AcrB
Brown			
*Laminaria japonica*	5.2	125	>500
*Sargassum horneri*	4.3	250	>500
Red			
*Gracilaria *sp.	6.3	62.5	>500
*Porphyra dentata*	3.9	250	>500

**Table 2:** Erythromycin-modulation activity of the seaweed extracts for Kam3 and Kam3-AcrB.

*E. coli*	Macroalgal extracts	Extracts conc.	IC_50_ of Erythromycin (*μ*g/mL)		Modulation factor
			No extract	With extract	
Kam3	*Laminaria japonica*	IC_50_/2	15.63	3.90	4
		IC_50_/4	15.63	15.63	1
	*Sargassum horneri*	IC_50_/2	15.63	3.90	4
		IC_50_/4	15.63	7.81	2
	*Gracilaria* sp.	IC_50_/2	15.63	3.90	4
		IC_50_/4	15.63	3.90	4
	*Porphyra dentata*	IC_50_/2	15.63	15.63	1
		IC_50_/4	15.63	NA	NA

Kam3-AcrB	*Laminaria japonica*	IC_50_/2	62.5	62.5	1
		IC_50_/4	62.5	62.5	1
	*Sargassum horneri*	IC_50_/2	62.5	7.81	8
		IC_50_/4	62.5	31.25	2
	*Gracilaria *sp.	IC_50_/2	62.5	31.25	2
		IC_50_/4	62.5	31.25	2
	*Porphyra dentata*	IC_50_/2	62.5	125	0.5
		IC_50_/4	62.5	62.5	1

NA, not applicable.

**Table 3:** Clarithromycin-modulation activity of the seaweed extracts for Kam3 and Kam3-AcrB.

*E. coli*	Macroalgal extracts	Extracts conc.	IC_50_ of Clarithromycin		Modulation factor
			No extract	With extract	
Kam3	*Laminaria japonica*	IC_50_/2	21.88	2.73	8
		IC_50_/4	21.88	21.88	1
	*Sargassum horneri*	IC_50_/2	21.88	2.73	8
		IC_50_/4	21.88	21.88	1
	*Gracilaria* sp.	IC_50_/2	21.88	2.73	8
		IC_50_/4	21.88	5.47	4
	*Porphyra dentata*	IC_50_/2	21.88	21.88	1
		IC_50_/4	21.88	NA	NA

Kam3-AcrB	*Laminaria japonica*	IC_50_/2	175	43.75	4
		IC_50_/4	175	87.5	2
	*Sargassum horneri*	IC_50_/2	175	10.94	16
		IC_50_/4	175	43.75	4
	*Gracilaria *sp.	IC_50_/2	175	87.5	2
		IC_50_/4	175	87.5	2
	*Porphyra dentata*	IC_50_/2	175	87.5	2
		IC_50_/4	175	87.5	2

NA, not applicable.

**Table 4:** Chemical composition of the ethanol extracts from the seaweeds.

Classifications	Brown seaweeds		Red seaweeds	
	*L. japnoica*	*S. horneri*	*Gracilaria* sp.	*P. dentata*
Terpenes	+	++	++	+
Terpenoids	+	+	+	+
Phenolic compounds	++	++	++	++
Indoles	–	+	–	+
Pyrrole derivatives	–	–	+	–
Halogenated aromatic compounds	–	–	+	+
Alkaloids	–	–	+	+

++, dominantly present in the extract; +, present in the extract; –, absent in the extract.

## Data Availability

The data used to support the findings of this study are included within the article.
